# Nucleation of the destruction complex on the centrosome accelerates degradation of β-catenin and regulates Wnt signal transmission

**DOI:** 10.1073/pnas.2204688119

**Published:** 2022-08-29

**Authors:** Ryan S. Lach, Chongxu Qiu, Erfan Zeyaei Kajbaf, Naomi Baxter, Dasol Han, Alex Wang, Hannah Lock, Orlando Chirikian, Beth Pruitt, Maxwell Z. Wilson

**Affiliations:** ^a^Department of Molecular, Cellular, and Developmental Biology, University of California, Santa Barbara, CA 93106;; ^b^Neuroscience Research Institute, University of California, Santa Barbara, CA 93106;; ^c^Biomolecular Science and Engineering, University of California, Santa Barbara, CA 93106;; ^d^Department of Mechanical Engineering, University of California, Santa Barbara, CA 93106;; ^e^Center for BioEngineering, University of California, Santa Barbara, CA 93106

**Keywords:** Wnt, destruction complex, optogenetics, LLPS, stem cells

## Abstract

Wnt signal transduction is controlled by the destruction complex (DC), a condensate comprising scaffold proteins and kinases that regulate β-catenin stability. Overexpressed DC scaffolds undergo liquid–liquid phase separation (LLPS), but DC mesoscale organization at endogenous expression levels and its role in β-catenin processing were previously unknown. Here, we find that DC LLPS is nucleated by the centrosome. Through a combination of CRISPR-engineered custom fluorescent tags, finite element simulations, and optogenetic tools that allow for manipulation of DC concentration and multivalency, we find that centrosomal nucleation drives processing of β-catenin by colocalizing DC components to a single reaction crucible. Enriching GSK3β partitioning on the centrosome controls β-catenin processing and prevents Wnt-driven embryonic stem cell differentiation to mesoderm. Our findings demonstrate the role of nucleators in controlling biomolecular condensates and suggest tight integration between Wnt signal transduction and the cell cycle.

## Significance

Liquid–liquid phase separation (LLPS) governs a variety of mesoscale cellular processes. However, less is known about how cells utilize LLPS to drive cellular function. Here, we examined the destruction complex (DC), an organelle which controls Wnt signaling and whose components phase separate. Through a combination of advanced microscopy, CRISPR, computational modeling, and optogenetics, we find that the DC is nucleated by the centrosome and that this nucleation drives efficient signal transduction. Our work not only uncovers a biological function for LLPS but also highlights nucleation as a general method for controlling the function of intracellular condensates. Finally, our findings suggest a thermodynamic coupling between Wnt signal transduction and the cell cycle which could lead to insights into Wnt-driven cancers.

The canonical Wnt signaling pathway is a conserved (1), morphogenic pathway that is essential for embryonic development, maintains adult tissue homeostasis, and, when dysregulated, induces malignancies (2–4). Wnt signals converge onto a protein assembly called the destruction complex (DC), which tunes the stability of β-catenin (β-cat), the pathway’s central transcriptional effector, by regulating its interactions with the kinases, CK1α and GSK3β, and the ubiquitinase, β-TRCP, which directs β-cat to the ubiquitin-mediated proteolysis machinery. Despite the DC’s role in regulating β-cat stability, the structural principles that underly its functioning in development and disease are still poorly understood.

Extracellular Wnt ligands inhibit DC function through a mechanism that is still unclear, but likely involves selective recruitment of DC components to the signalosome, a biomolecular condensate on the plasma membrane that includes Wnt/Frizzled/LRP5/6 clusters (5). Optogenetic clustering of LRP5/6 is sufficient to stabilize β-cat (6), suggesting the formation of mesoscale protein clusters at the Wnt receptor level is necessary and sufficient for activating the pathway. Less is known about the DC’s native structure and how it maintains low β-cat levels in the Wnt OFF state. Recently, the DC scaffolds Axin and Adenomatous Polyposis Coli (APC), and the signalosome “adapter” protein Disheveled have been shown to undergo liquid–liquid phase separation (LLPS) in vitro (7) and, when exogenously overexpressed, in vivo (8–10). Cancer-causing mutations that eliminate Axin or APC LLPS are correlated with aberrant accumulation of β-cat (11, 12) and can be rescued by orthogonal protein-multimerizing domains (13). These studies raise the question, What is the role of mesoscale assembly of the cytoplasmic DC components in regulating β-cat stability?

The “molecular crucible” model posits that DC LLPS promotes β-cat degradation in Wnt OFF conditions (7) via concentration of DC clients (CK1α, GSK3β, and β-cat) in DC scaffold (Axin and APC) condensates to increase the rate of β-cat processing. LLPS-mediated DC concentration is distinct from theories suggesting that the DC acts as an ordered, assembly line–like scaffold akin to Ste5 in the yeast MAPK pathway (14). Indeed, deletions in Axin regions that promote LLPS (15) increase β-cat stability (7). In this paradigm, conditions that alter the phase behavior of scaffolds and partitioning of clients are predicted to regulate the stability of β-cat and Wnt signal transmission.

A biophysical mechanism for regulating condensates is control over their nucleation. This principle was recently explored with a synthetic optogenetic system (16), but the role of nucleation in natural biological processes remains unknown. Phase-separating systems exhibit switch-like responses to changes in concentration (17, 18) and often exist near these transitions in vivo (19, 20). Dissecting the mechanisms controlling LLPS requires control over protein concentration and affinity, and overexpression of DC components may not recapitulate endogenous mesoscale structure. Here, we utilize CRISPR gene editing, custom inducible expression vectors, and optogenetic tools to observe and probe the native, mesoscale organization of the DC in the Wnt OFF and Wnt ON states.

Building on results demonstrating that β-cat (21, 22), Axin1 (23), APC (24), and βTrCP/*Slimb* (25) localize to the centrosome, we show that all DC components are nucleated by the centrosome into liquid-like biomolecular condensates. In support of the molecular crucible theory, we find that nucleation drives efficient degradation of β-cat. We utilize a Cahn–Hilliard-based simulation of DC droplet formation and enzyme kinetics to predict how nucleation and affinity of DC components promotes efficient β-cat processing. Finally, using our model as a guide, we engineered a light-inducible GSK3β (Opto-GSK3) to control partitioning at the centrosome, β-cat degradation, and stem cell differentiation into mesoderm. These findings show that DC droplet formation is nucleated by the centrosome and suggest that DC scaffolds function to concentrate clients in liquid droplets in vivo to accelerate the degradation of β-cat.

## Results

### β-Cat Condensation Is Predictive of Wnt Pathway Activity State.

**Fig. 1.**
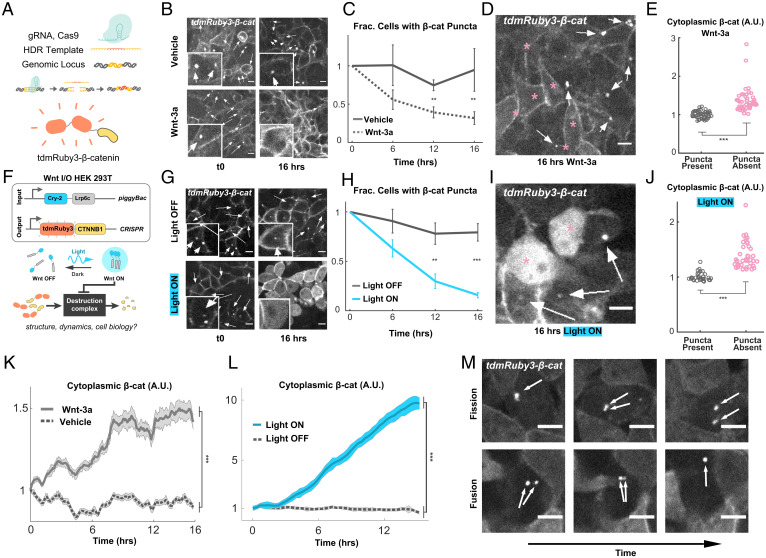
Endogenously expressed β-cat puncta are inversely correlated with lrp6-mediated Wnt pathway activation and β-cat accumulation. (*A*) Schematic of tdmRuby3 CRISPR tag strategy. (*B*) Representative tdmRuby3-β-cat images of cells treated with Wnt-3a or media vehicle. Arrows indicate β-cat puncta. (Scale bars, 10 μm.). Insets show closeup examples of presence (arrows) or lack of puncta. (*C*) Fraction of t0 population with visible β-cat puncta, presented as mean ± SEM (*n* = 12 imaging fields per condition). (*D*) Representative cells from Wnt-3a condition. Arrows indicate puncta, and asterisks indicate cells lacking puncta. (Scale bars, 10 μm.) (*E*) Comparison of mean cytoplasmic β-cat fluorescence between Wnt-3a cells with and without visible β-cat puncta. (*F*) Schematic of Wnt I/O cells containing lentivirally expressed Cry2-LRP6c and CRISPR-tagged tdmRuby3-β-cat. Stimulation of Cry-2-Lrp6c with blue light results in reversible clustering of lrp6c and downstream pathway activation. (*G*) Representative tdmRuby3-β-cat images of cells stimulated with blue light or left in the dark throughout imaging time course. Insets show closeup examples of presence (arrows) or lack of puncta. (Scale bars, 10 μm.) (*H*) Fraction of t0 population with visible β-cat puncta, presented as mean ± SEM (*n* = 12 imaging fields per condition). (*I*) Representative cells from Wnt-3a condition. Arrows indicate puncta, and asterisks indicate cells lacking puncta. (Scale bars, 10 μm.) (*J*) Comparison of mean cytoplasmic β-cat fluorescence between Light ON cells with and without visible β-cat puncta. (*K* and *L*) Measurements of CRISPR cytoplasmic tdmRuby3-β-cat in live 293Ts; data presented as mean fluorescent intensity fraction of t0 ± SEM (*n* = 30 cells per condition). (*M*) Time course montage of single CHIR+ cells containing β-cat puncta undergoing dynamic fission and fusion. Arrows indicate puncta. Images are from consecutive frames of time course, separated by 5-min intervals. (Scale bars, 10 μm.)

To understand the role of mesoscale organization in DC function, we first sought to characterize the DC’s main substrate, β-cat in live cells. We used CRISPR to knock in a custom fluorescent tag, tdmRuby3, to the *CTNNB1* gene of 293T cells (Fig. 1*A*). Live-cell confocal imaging revealed expected cytoplasmic accumulation in response to Wnt-3a ligand and the GSK3β inhibitor CHIR (*SI Appendix*, Fig. S1 *A* and *B* and Movie S1) and localization of β-cat at the cell membrane, consistent with previous work in fixed specimens (26). In addition, we observed that most cells contained one or two bright, spherical, perinuclear β-cat puncta (Fig. 1 *B*). Timelapses showed fission and fusion of puncta on the timescale of minutes (Fig. 1*M*), suggesting that these structures are liquid-like biomolecular condensates. Given the prevalence of biomolecular condensates in organizing important biological processes, we hypothesized that these perinuclear puncta might organize β-cat destruction.

To determine whether Wnt pathway activation altered the perinuclear puncta, we performed volumetric confocal timelapse microscopy on our *tdmRuby3-β-cat* cells and quantified the fraction of cells with puncta as a function of Wnt-3a ligand treatment and time. At the population level, the fraction of cells with puncta significantly decreased in response to Wnt-3a (Fig. 1*C*). We found this same relationship existed between single cells in an isogenic population: nonresponding cells maintaining their puncta and responding cells dissolving them (Fig. 1 *D* and *E*). Thus, the disappearance of perinuclear β-cat puncta is correlated with β-cat accumulation, and the existence of these puncta is correlated to the resistance of ligand-induced accumulation.

To establish whether directly activating the Wnt receptor controls the existence of the puncta, we transduced *tdmRuby3-β-cat* cells with an optogenetic version of the Wnt coreceptor, LRP6c (Opto-LRP6) (6). Opto-LRP6 induced greater accumulation of β-cat than either Wnt or CHIR (Fig. 1 *K* and *L*). We thus reasoned that this all-optical Wnt input control and output visualization cell line would maximize our ability to observe rearrangements in pathway components due to a higher dynamic range of activation (Fig. 1*F*). We found that activating Opto-LRP6 resulted in a greater reduction in the fraction of cells containing β-cat puncta than treating cells with ligand (Fig. 1 *G* and *H* and Movies S2 and S3). β-cat puncta became more difficult to distinguish at higher cytoplasmic concentrations produced by activated Opto-LRP6, but dissolution nearly always preceded appreciable dilute-phase β-cat accumulation, indicating that they were not simply obscured by higher background levels. Further, of light-stimulated cells, those that were resistant to optogenetic activation maintained their β-cat puncta (Fig. 1 *I* and *J*). We also observed this same resistance to β-cat accumulation in response to CHIR (*SI Appendix*, Fig. S1*B*). Together, these results indicate that activation of the Wnt pathway causes perinuclear puncta to dissolve, and the presence of these puncta is inversely related to Wnt pathway activation at the population and single-cell levels.

### The DC Forms a Biomolecular Condensate Colocalized to the Centrosome.

We next sought to determine 1) what, if any, cellular structure was organizing these puncta, 2) whether all DC components were colocalized with puncta, and 3) whether these were solid or liquid-like condensates. Because of the sensitivity of LLPS systems to protein concentration (27), we decided on a strategy that allowed for visualization of DC components at low or endogenous concentrations, while retaining the ability to assess protein dynamics through live-cell microscopy and fluorescence recovery after photobleaching (FRAP). Indeed, DC scaffolds APC and Axin1 form multiple liquid droplets when overexpressed (12, 28). Thus, we used CRISPR to knock in tdmRuby3 into the loci of *CSNK1A1*, *GSK3B,* and *AXIN1*, genes encoding the kinases CK1α and GSK3β that sequentially phosphorylate β-cat in the DC, and the primary DC scaffold.

**Fig. 2.**
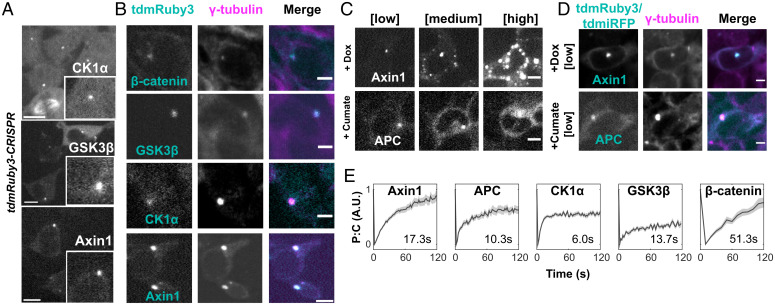
Canonical DC components reside in liquid droplets nucleated at the centrosome. (*A*) Representative images of CRISPR-integrated tdmRuby3-CK1α, tdmRuby3-GSK3β, and Axin1-tdmRuby3 cells. (*Insets*) Close-up views of singular perinuclear puncta. (Scale bar, 10 μm.) (*B*) Representative cells bearing the indicated DC component fixed and stained for endogenous γ-tubulin. (Scale bar, 10 μm.) (*C*) Representative timelapse images from live cells bearing dox- and cumate-inducible Axin1 and APC cassettes under induction. Montages depict the same cell increasing its DC scaffold concentration through time. (Scale bar, 10 μm.) (*D*) Representative cells bearing the indicated DC component fixed and stained for endogenous γ-tubulin. (Scale bar, 10 μm.) (*E*) FRAP traces of mean puncta:cytoplasm fluorescence ratio for indicated DC components. Data are presented as mean ± SEM normalized to extent of bleaching (*n* = 39, 20, 33, 17, and 22 for Axin1, APC, CK1α, GSK3β, and β-cat, respectively). Individual FRAP traces were fit to the equation: f(t) = *a*(1-e^(-*b*t)^) to obtain a and b parameters and half-max recovery time (τ1/2). Mean τ1/2 for each DC component is displayed on each plot.

We found that all tagged proteins were localized into one or two perinuclear puncta (Fig. 2*A*). Timelapses revealed that the number and position of the puncta were determined by cell cycle stage (*SI Appendix*, Fig. S2*A*): We observed single condensates in G1, two condensates in G2/S, and a “finger-like” pattern—suggesting association with the mitotic spindle—during late mitosis. These observations, combined with previous reports of perinuclear enrichment of CK1α, GSK3β, and Axin1 in fixed cells (29–31), led us to hypothesize that these DC components and β-cat were associated with the centrosome. Immunofluorescence staining for γ-tubulin (Fig. 2*B*) and GM130 (*SI Appendix*, Fig. S2*B*) confirmed that tdmRuby3-CK1α, tdmRuby3-GSK3β, and tdmRuby3-β-cat puncta were indeed colocalized to the centrosome.

When overexpressed, Axin and APC cross the phase boundary and form liquid condensates in the cytoplasm that are hypothesized to concentrate DC kinases and β-cat (32). The fact that no extracentrosomal DC puncta were observed in cells at endogenous concentrations led us to hypothesize that the DC is a liquid organelle that is nucleated at the centrosome at endogenous protein concentrations, but forms extracentrosomal condensates at higher concentrations. To test whether Axin1 and APC are localized to the centrosome at low cellular concentrations, but not when overexpressed, we generated clonal 293Ts bearing doxycycline (Dox)-inducible human Axin1-tdmRuby3 and cumate-inducible human APC-tdmiRFP670. At low levels of induction, both Axin1 and APC localization mirrored CRISPR CK1α, GSK3β, Axin1, and β-cat, forming bright perinuclear puncta (Fig. 2*C*, *Left*) that colocalized with centrosomal markers (Fig. 2*D* and *SI Appendix*, Fig. S2*B*) and replicated following cell cycle progression (*SI Appendix*, Fig. S2*A*). As protein concentration increased, Axin1, but not APC, caused formation of extracentrosomal puncta throughout the cytoplasm (Fig. 2 *C–E*). To determine whether extracentrosomal condensates observed at high Axin1 concentrations were capable of concentrating canonical DC components similar to centrosomal DCs, we next expressed Dox-Axin1-GFP in CRISPR tdmRuby3-CK1α and GSK3β backgrounds. Cells with high Axin1 levels formed extracentrosomal condensates colocalized with APC, CK1α, and β-cat (*SI Appendix*, Fig. S2*E*). Interestingly, extracentrosomal Axin1 condensates did not reliably induce formation of extracentrosomal GSK3β condensates in these experiments, but often resulted in deenrichment of centrosomal puncta (*SI Appendix*, Fig. S2*E*). We reason that this was due to extracentrosomal Axin1 condensates competing for relatively scarce GSK3β, thereby diluting across all condensates in the cytoplasm.

The 293Ts are commonly used in experiments probing DC mesoscale structure in vivo (7, 10), but expression of Wnt pathway components may vary significantly between stem cells and differentiated cells. We observed the same preferential localization of Axin1 at low concentration in human induced pluripotent stem cells(hiPSCs) (*SI Appendix*, Fig. S2*F*). These findings establish that all DC components necessary for phosphorylating β-cat, prior to its ubiquitination, are localized at the centrosome throughout the cell cycle and suggest that DC centrosomal nucleation is generalizable to multiple cell types.

Next, we sought to determine the material state of the centrosomal DC using FRAP on CRISPR-tagged CK1α, GSK3β, and β-cat, as well as of Axin1 and APC at low levels of induction. All centrosomal DC components exhibited mean half-maximal recovery times (τ1/2) between 10 s and 60 s (Fig. 2*E*)—like in overexpressed systems (15) and in line with mesoscale cellular structures considered liquid-like (33). Interestingly, relatively wide variation in both stable fraction and τ1/2 was observed between centrosomal DC components, indicating differential turnover of monomers between condensates and the bulk cytoplasm. This suggests that multiple biophysically distinct pools of each component, with different condensation dynamics, coexist at the centrosome together. Despite this, these results support the idea that the DC is a liquid nucleated by the centrosome and suggest that nucleation has a role in maintenance of cellular β-cat levels.

### A Reactive Cahn–Hilliard Model Predicts Accelerated β-Cat Processing upon Centrosomal Nucleation of DC Clients.

**Fig. 3.**
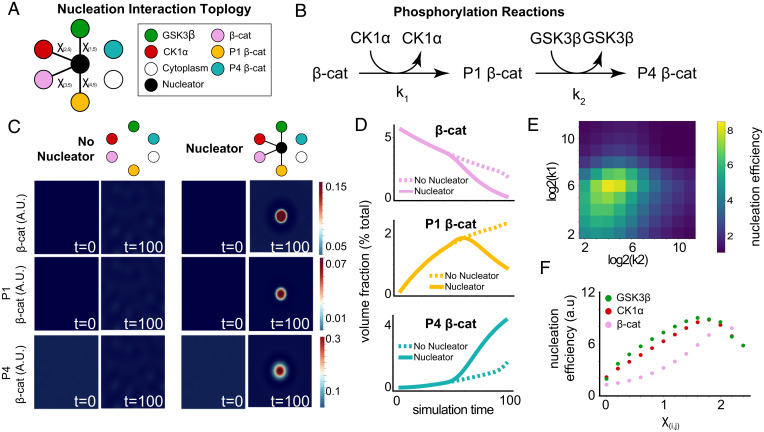
In silico modeling of β-cat processing efficiency from a nucleated liquid droplet. (*A*) Nucleation interaction topology that describes the pairwise interactions between each component of the simulation. Connected components minimize free energy by mixing, and unconnected components either demix or remain in a noninteracting neutral state. (*B*) Schema describing the phosphorylation reactions and rates modeled in the simulation. (*C*) Simulation at steps 0 and 100 comparing a system with and without a centrosome. (*D*) Quantification of each form of β-cat with and without a centrosome. (*E*) Nucleation efficiency as a function of both rate parameters k_1_ and k_2_. (*F*) Nucleation efficiency in simulations as a function of the interaction parameters between a single client and the cytoplasm.

To understand the effect of centrosomal nucleation of DC components on β-cat processing, we simulated the processive phosphorylation of β-cat by DC kinases, using a reactive, multicomponent, Cahn–Hilliard system (34, 35). We represented the function of DC scaffolds implicitly through the interaction parameters between kinases and β-cat (Fig. 3 *A* and *B* and *SI Appendix*, Fig. S3 *A–D*). Indeed, synthetic DC scaffolds with these simple attributes have been shown to rescue aberrant Wnt signaling (36).

To test the effects of nucleation on β-cat processing, we compared simulations in the presence and absence of a nucleation region (Fig. 3*C*). We found that, for systems that did not spontaneously phase separate, mimicking the endogenously expressed conditions observed above, DC components localized into a single droplet surrounding the nucleator but did not spontaneously demix in its absence (Movies S4 and S5). We found that the nucleated system processed β-cat and its intermediates more quickly (Fig. 3*D*) over a wide range of nucleator sizes (*SI Appendix*, Fig. S3*E*). See *Materials and Methods* for a detailed discussion of nucleation parameter scan results. Notably, the nucleated system accelerated β-cat processing, increasing pathway efficiency (*SI Appendix*, Fig. S3*F*). This efficiency gain was maintained over a large range of reaction rates (Fig. 3*E* and *SI Appendix*, Fig. S3*G*). As expected, in systems with high reaction rates, the effect of nucleated phase separation is no longer observed.

Given our findings that nucleation drives efficient processing of β-cat, we hypothesized that χ, the interaction parameter that drives phase separation, is a control parameter for β-cat processing. To determine the relationship between DC function and the interaction strength parameter, we systematically decreased the χ between DC clients and the cytoplasm. We found that reducing condensation on the nucleator, through altering χ, decreased the speed and efficiency of β-cat processing (*SI Appendix*, Fig. S3 *H* and *I*, Fig. 3*F*, and Movie S6). Together, these results demonstrate that nucleation of DC components has the potential to increase β-cat processing and that a tunable control parameter of this process is the free energy of mixing.

### Optogenetically Driven Enrichment of Centrosomal GSK3β Condensates Rescues Hyperactivated Wnt Signaling.

In silico analysis of the DC indicates that processing efficiency in the presence of a nucleator is dependent on client condensation. Imaging of GSK3β showed relatively weak enrichment in centrosomal puncta compared to CK1α, suggesting that increasing nucleation of GSK3β would increase the degradation rate of β-cat in vivo. Changing concentration alters both propensity to undergo LLPS and reaction rate (37) and therefore cannot be used to test the effect of nucleation on reaction rate. Optogenetic photoclustering domains can independently control intracellular LLPS at fixed concentrations via light-induced changes in valency between monomers (38, 39). Thus, we reasoned that an optogenetic tool that drives changes in free energy could isolate the effect of nucleation from biological function.

**Fig. 4.**
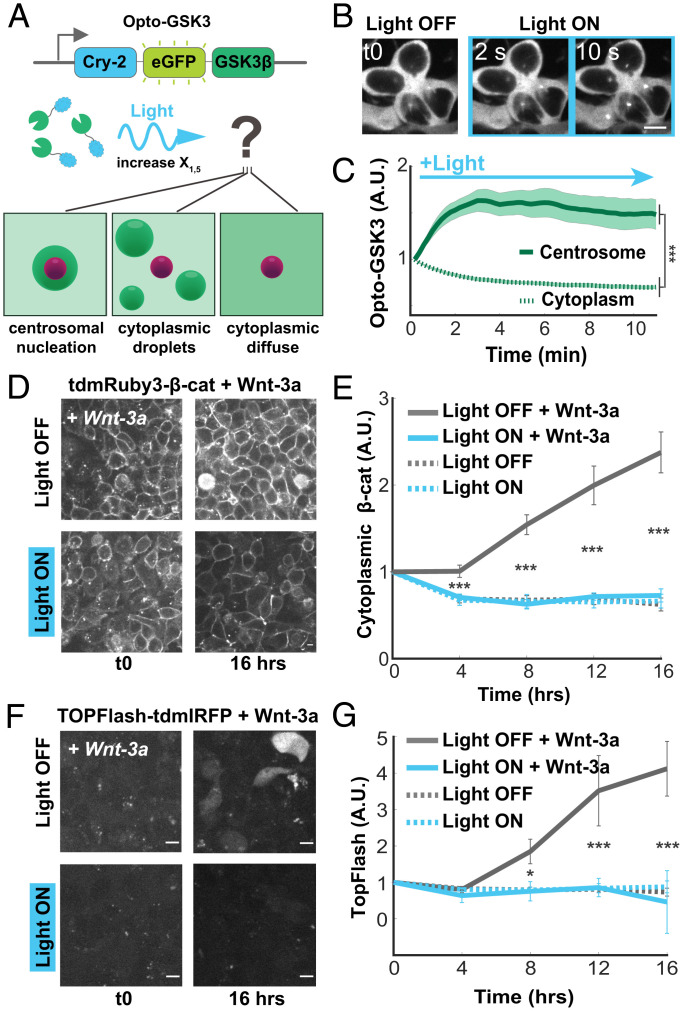
Optogenetic clustering of GSK3β increases centrosomal droplet partitioning and suppresses Wnt pathway activation. (*A*) Schematic of Opto-GSK3 and possible spatial outcomes of blue light stimulation. (*B*) Representative images of cells bearing Opto-GSK3 responding to blue light stimulation. Montage depicts the same cells throughout the activation time course. (Scale bar, 10 μm.) (*C*) Quantification of cells in *B*. Mean fluorescence fold change from t0 for each compartment ± SEM (*n* = 20 cells). (*D*) Representative images of cells bearing Opto-GSK3 + tdmRuby3-β-cat following treatment with Wnt-3a. (Scale bar, 10 μm.) (*E*) Quantification of cells in *D*. Mean fluorescence fold change from t0 ± SEM is shown (*n* = 20 cells per condition). (*F*) Representative images of cells bearing Opto-GSK3 + TOPFlash-IRFP following treatment with Wnt-3a. (Scale bar, 10 μm.) (*G*) Quantification of *F*. Mean fluorescence fold change from t0 ± SEM is shown (*n* = 24 cells per condition).

To test whether photoclustering increases partitioning to a nucleator, we fused the photooligomerizer Cryptochrome-2 (Cry-2) and eGFP to human GSK3β (“Opto-GSK3” hereafter) and stably transduced it into 293Ts (Fig. 4*A*). Upon light stimulation, Opto-GSK3 increased its centrosomal enrichment, doubling the mean centrosome:cytoplasm fluorescence ratio within 10 s of activation (Fig. 4 *B* and *C* and Movie S7). Notably, activation of Opto-GSK3 strictly resulted in the formation of one or two perinuclear puncta and did not form extracentrosomal condensates, contrasting with results from studies using Cry2 alone (17). Thus, we found that illumination of Opto-GSK induced condensate formation only at the centrosome.

To determine whether increased centrosomal condensation of GSK3β controlled Wnt signal transmission, we activated Opto-GSK3 in cell lines with three distinct methods for increasing the cellular concentration of β-cat: ligand-induced, kinase inhibition, and dox-induced gene up-regulation. We found that Opto-GSK activation abolished both Wnt-3a-induced β-cat accumulation and transcriptional activation as measured by TOPFlash fluorescence (Fig. 4 *D* and *E*). Control experiments comparing cells in light vs. dark confirmed that this was not due to light alone (*SI Appendix*, Fig. S4 *A* and *B*). We observed a similar effect when analyzing total β-cat by Western blotting and immunofluorescence staining (*SI Appendix*, Fig. S4 *C–E*). Given the modest accumulation of β-cat in response to Wnt-3a in 293Ts, we tested to see whether Opto-GSK3 clustering was sufficient to blunt β-cat accumulation induced by either CHIR or a Dox-inducible β-cat overexpression construct. Indeed, activation of Opto-GSK3 also inhibited both methods for driving β-cat accumulation in a light-dependent manner (*SI Appendix*, Fig. S4 *F*–*J*). These results demonstrate that increasing DC client nucleation at the centrosome dictates Wnt signal transmission across a wide range of activation regimes.

### Centrosomal Enrichment of GSK3β Prevents Wnt Pathway Activation-Induced Differentiation of Embryonic Stem Cells.

**Fig. 5.**
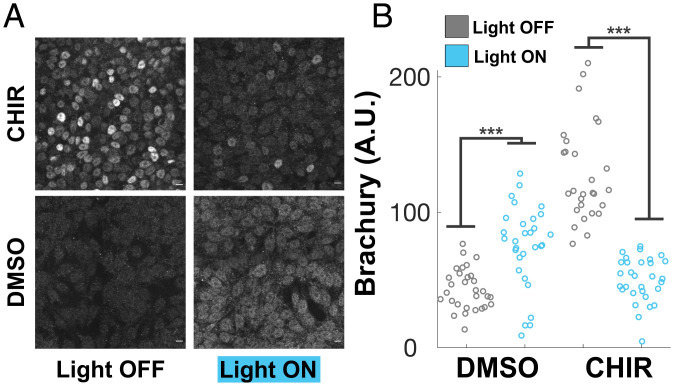
Optogenetic clustering of GSK3β suppresses Wnt pathway-mediated differentiation of embryonic stem cells. (*A*) Representative images of H9 embryonic stem cells bearing Opto-GSK3 following 24 h in described conditions, fixed and stained for endogenous Brachyury. (*B*) Quantification of experiment from *A*. Mean nuclear fluorescence for cells measured in each condition is presented.

Changes in β-cat concentration differentiate a variety of stem cell populations, including human embryonic stem cells (hESCs) (38, 40). Having determined that increased centrosomal nucleation of GSK3β is sufficient to reduce β-cat accumulation and Wnt-responsive gene transcription in 293T cells, we wondered whether it was also sufficient to prevent the downstream differentiation of hESCs. Both CHIR and Wnt-3a induce hESC differentiation into mesoderm (41). To test whether centrosomal nucleation prevents differentiation, we expressed Opto-GSK3 in H9 hESCs and treated them with CHIR or dimethyl sulfoxide (DMSO) control in the presence or absence of activating blue light. Following stimulation, cells were fixed and stained for Brachyury (BRA) to assay for differentiation. In the dark, hESCs receiving CHIR responded robustly, displaying bright nuclear BRA compared to DMSO controls (Fig. 5 *A*). However, when stimulated with blue light, CHIR-treated cells showed significantly reduced levels of BRA staining compared to the dark controls, indicating that nucleation of GSK3b countered CHIR-induced differentiation into mesoderm (Fig. 5 *A*). Interestingly, we observed that BRA levels in DMSO and light condition were slightly, but significantly, higher than when in the dark, suggesting that overrepression of the Wnt pathway by Opto-GSK3 activation weakly promotes differentiation in hESCs as well (Fig. 5*B*).

## Discussion

Building on recent discoveries suggesting that LLPS plays a role in DC structure, we sought to understand how the biophysics of DC proteins regulate DC function in live cells. Through a combination of superresolution microscopy, in silico modeling, and optogenetic methods to isolate and probe the phase diagram, we discovered that the mesoscale structure of the DC is a liquid condensate nucleated by the centrosome. The complementarity of these methods allowed us to identify a function for nucleation: acceleration of the catalytic action of DC proteins, thereby promoting efficient processing of β-cat.

The presence of many cytoplasmic Axin1 and APC droplets in mildly overexpressed cellular conditions (9) has been cited in support of the idea that DC scaffolds spontaneously phase separate at endogenous concentrations. Yet, because of the sensitivity of LLPS to concentration, we sought to examine the biophysics of DC components at endogenous concentrations. We found that, at low or endogenous levels, all DC components form dynamic assemblies with preferred localization to the centrosome. These results suggest that centrosomal nucleation lowers the concentration threshold for DC condensation.

Our results support a “molecular crucible” model of β-cat degradation, in which multivalent DC scaffolds concentrate DC clients in nucleated droplets to increase β-cat phosphorylation rate. Assembly line models for β-cat degradation have been proposed (15, 42), and Axin1 polymerization has been observed to be ordered in vitro (28). Yet, others have shown that DC condensates display hallmarks of disorder, such as surface tension minimization, rapid fission/fusion (43), and responsivity to concentration and interaction strength (9). Our results demonstrate that increased multivalency due to optogenetic photoclustering accelerates β-cat degradation, suggesting that DC function is responsive to disordered partitioning of DC clients into condensates.

We found that centrosomal DCs cease to concentrate β-cat under Wnt ON and GSK3β chemical inhibition, but the mechanism for this change remains unclear. Multiple DC components that bind β-cat—including Axin1, GSK3β, and CK1α—are also binding partners of the Frizzled-LRP6 signalosome (44, 45), a known inhibitor of GSK3β’s phosphorylation of β-cat. Wnt-activated signalosomes may therefore compete with β-cat and/or GSK3β for DC proteins necessary for phosphorylation and degradation, resulting in the accumulation of nascent β-cat in the cytoplasm. For example, GSK3β phosphorylation is known to regulate APC’s R2/B motif, which is critical for APC/Axin interaction and β-cat degradation (15). Alternatively, Dvl was recently found to regulate Wnt pathway activation via its affinity for Axin1’s DIX domain (13), potentially “invading” and destabilizing Axin–Axin multimerization; such invasion of the DC could dilute Axin1 and its associated clients in the DC, reducing the phosphorylation rate of β-cat.

Our results raise an important question that may lead to the discovery of unknown potentiators of Wnt signal transduction: What is/are the nucleator(s) coupling the DC to the centrosome? Axin1 is known to associate with γ-tubulin (31) and is a substrate of PLK1, a kinase involved in centrosome duplication during cell cycle progression (23), suggesting that it is redundantly associated with the centrosome. APC is a regulator of microtubule stability and growth (24, 46), and its armadillo repeat region is sufficient to induce centrosomal localization (24). Multiple binding sites for DC scaffolds could localize the DC to the centrosome, increasing the robustness of droplet nucleation and enriching local client concentration. Notably, elimination of centrioles in developing mice and *Drosophila* embryos leads to only minor tissue-level defects in canonical Wnt signaling and overall morphology (47, 48), indicating that centrosomes are not essential for Wnt-mediated embryogenesis. We show that Axin is critically poised at the phase boundary, so it is possible that DC condensation is restored via simple up-regulation of this scaffold or the presentation of another nucleator through feedback mechanisms. Alternatively, another undiscovered DC nucleator that normally localizes to the centrosome may be sufficient to drive DC condensation when the centrosome is absent.

Finally, centrosomal nucleation of the DC suggests a potential function in coordinating cell cycle progression with Wnt signaling. We found two DC droplets in cells with duplicated centrosomes, suggesting that the DC is split along with centrosomes during mitosis. Nonnucleated droplets would be randomly partitioned into daughter cells, leading to potentially detrimental asymmetry in Wnt signaling capacity of the growing tissue. Cell cycle synchronization could be a method of reducing heterogeneity in Wnt-induced stem cell differentiations.

Overall, our studies suggest an integral role for LLPS nucleation in regulating the activity of membraneless organelles in vivo. The power of observing proteins in their endogenous contexts, coupled with the ability to precisely tune interaction strength without altering protein function or concentration, enables the functional dissection of membraneless organelles.

## Materials and Methods

### Cell Lines.

Human 293T cells were cultured at 37 °C and 5% CO_2_ Dulbecco’s Modified Eagle Medium, high glucose GlutaMAX (Thermo Fisher Scientific, 10566016) medium supplemented with 10% fetal bovine serum (Atlas Biologicals, F-0500-D), and 1% penicillin-streptomycin. The hiPSC WTC was gifted by the B.P. laboratory (purchased from Coriell). The hiPSCs were propagated on Matrigel-coated tissue culture plates using serum-free essential 8 (Gibco) culture conditions in standard environments consisting of 5% carbon dioxide at 37 °C. Experiments in hESC lines were performed using the H9 hESC cell line purchased from the William K. Bowes Center for Stem Cell Biology and Engineering at University of California, Santa Barbara (UCSB). Cells were grown in mTeSR Plus medium (Stem Cell Technologies) on Matrigel (Corning)-coated tissue culture dishes and tested for mycoplasma in 2-mo intervals.

### Cloning of PiggyBac Transposase and Lentiviral Overexpression Constructs.

The pPig_H2B-mTagBFP2::t2A::Cas9-Avidin was constructed via subcloning human H2B, mTagBFP2, and Cas9-Avidin provided by M.Z.W. into an expression vector bearing a cytomegalovirus CMV promoter and flanking PiggyBac transposase-compatible inverted terminal repeats using Gibson Assembly (New England BioLabs Inc., E2611L) according to supplier instructions. Each of the PCR fragments used was amplified using the following primers:

PiggyBac (CMV) Backbone fwd: tgacgcccgccccac rev: ggtaagctttttgcaaaagcctaggcc. H2B + 18AA linker fwd: cctaggcttttgcaaaaagcttaccatgccagagccagcgaagtc rev: GCATATTTTCCTTGATGAGTTCACTCATccCagTatGtcCgcCggAg. mTagBFP2 fwd: ATGAGTGAACTCATCAAGGAAAATATGCACATG rev: CGTCCCCGCAGGTCAACAAACTTCCGCGACCTTCTCCGCTCCCATTGAGCTTATGGCCGAGTTTGCTG.-3′X-Flag-NLS-Cas9-HA-Avidin fwd: GGAAGTTTGTTGACCTGCGGGGACGTGGAAGAAAACCCGGGTCCAgactataaggaccacgacggagactac rev: gctgcgggtcgtggggcgggcgtcaggatccagacgccgcag

XLone-Axin-tdmRuby3 was constructed via PCR and Gibson Assembly, subcloning from the following constructs: Flag-Axin1 purchased from Addgene (#109370), tdmRuby3 from M.Z.W. into XLone-GFP purchased from Addgene (#96930) containing-3′^rd^ gen tet ON-responsive promoter, and EF1α-driven Blasticidin selection cassette. The following primers were used: XLone Backbone fwd: taaactagtagaccacctcccctgcg, rev: ggtacctttacgagggtaggaagtgg, human Axin1 fwd: cacttcctaccctcgtaaaggtaccatgaatatccaagagcagggtttcccc, rev: CCATgctTCCgCCgCCACTACCgCCgtccaccttctccactttgccgatgatc, 7AA link- tdmRuby3 fwd: GGcGGTAGTGGcGGcGGAagcATGGTTAGCAAAGGGGAGGAGC, rev: gcaggggaggtggtctactagtttaCTTGTACAGCTCGTCCATGCCG

XLone-bCat-tdmRuby3 was constructed via PCR and Gibson Assembly, subcloning from the following constructs: XLone-Axin-tdmRuby3 (above) and Human Beta-catenin GFP purchased from Addgene (#71367). The following primers were used: XLone Backbone fwd: GGcGGTAGTGGcGGcGGAagcATGGTTAGCAAAGGGGAGGAGC, rev: ggtacctttacgagggtaggaagtgg, human bcat fwd: cacttcctaccctcgtaaaggtaccatggctactcaagctgatttgatggagttg, rev: CCATgctTCCgCCgCCACTACCgCCcaggtcagtatcaaaccaggccagc

The pPig_CuO-APC-tdmIRFP670::CymR was constructed via PCR and Gibson Assembly from the following constructs: pCuo CA Rac1 CMV + cumate operon purchased from Addgene (#84643), human APC open reading frame (ORF) purchased from Addgene (#16507), tdmirfp670nano from M.Z.W., and human ubiquitin C-driven CymR Cuo repressor purchased from Addgene (#119907) into pPig-Hygro transposase backbone from M.Z.W. PCR fragments were amplified using the following primers:

pPig-Hygro Backbone fwd: GGACGTGGAAGAAAACCCGGGTCCAatgggtaaaaagcctgaactcaccgc, rev: cattccacagggtcgacagtacaagc,
Cuo + CMV fwd: cttgtactgtcgaccctgtggaatgcgttacataacttacggtaaatggcccgc,
rev: actgatcatatgaagctgcagccatgaattcggtaccggatccagtcgactag,
APC fwd: atggctgcagcttcatatgatcagttgttaaagcaag,
rev: CCATgctTCCgCCgCCACTACCgCCaacagatgtcacaaggtaagacccagaatg,
7AAlinker-tdmirfp670nano fwd: GGcGGTAGTGGcGGc,
rev: ggcgccaaaacccggcgcggaggccttaGGACTGCTGTATTGCAATGCCAACTAC,
UbC-CymR-V5-T2A fwd: ggcctccgcgccggg,
rev: TGGACCCGGGTTTTCTTCCACGTCCCCGCAGGTCAACAAACTTCCGCGACCTTCTCCGCTCCCcgtagaatcgagaccgaggagagg

The pLV_Cry2-tdeGFP-GSK3b was obtained via synthesis and cloning services provided by Vector Builder Inc. Full details are available upon request, but, briefly: primary plasmids containing *Arabadopsis thaliana*, tdmIRFP from M.Z.W. and human GSK3β purchased from Addgene (#16260) ORFs were supplied to VectorBuilder for cloning and EF1α-driven expression into third-generation lentiviral backbone. Vectorbuilder provided the desired final, sequenced plasmid.

The pPig_8XTOP_tdIRFP_Puro was constructed via PCR and Gibson Assembly from the following constructs: pPig_H2B-mTagBFP2::t2A::Cas9-Avidin (above), M50 Super 8× TOPFlash purchased from Addgene (#12456), and codon-optimized tandem (td) IRFP ordered from Twist Biosciences as overlapping gene fragments with the sequences:

ATGGCTGAAGGCAGCGTGGCCCGACAGCCAGACCTTTTGACTTGTGACGATGAACCAATCCACATACCGGGGGCAATACAACCTCATGGTCTCCTTCTGGCGCTTGCTGCCGACATGACTATAGTGGCCGGCTCTGACAACTTGCCGGAATTGACCGGACTTGCTATTGGGGCGTTGATTGGGCGCTCTGCCGCTGATGTATTTGATTCCGAGACACATAATAGGCTTACTATAGCCCTCGCCGAACCAGGGGCTGCCGTCGGCGCTCCTATAACAGTTGGGTTCACGATGCGAAAAGATGCTGGGTTCATTGGTAGCTGGCATCGCCACGATCAACTTATCTTCCTTGAGCTTGAACCCCCTCAACGGGACGTTGCGGAACCCCAAGCTTTCTTTAGAAGGACCAATTCAGCCATAAGGCGCCTTCAGGCCGCAGAGACATTGGAGTCCGCGTGTGCGGCAGCAGCGCAGGAAGTACGAAAGATCACGGGATTTGACCGGGTTATGATTTACAGATTCGCATCTGATTTCTCCGGGGAAGTCATCGCGGAGGATCGGTGTGCAGAAGTGGAAAGCAAGCTTGGTTTGCATTACCCCGCATCTACGGTTCCGGCCCAAGCGAGGAGACTGTATACGATAAACCCAGTGAGGATCATACCTGACATAAATTATAGACCGGTTCCCGTTACGCCAGACCTGAACCCCGTCACAGGCAGGCCAATAGACTTGTCTTTTGCAATCCTGCGGTCAGTCTCACCTGTTCACCTCGAGTTTATGAGGAACATAGGGATGCATGGGACGATGAGCATCTCAATCCTGAGAGGTGAACGGCTCTGGGGACTTATTGTTTGTCATCATCGCACACCGTATTACGTTGACCTTGATGGTCGCCAGGCCTGCGAACTCGTAGCTCAAGTATTGGCCTGGCAGATCGGTGTTATGGAGGAAAGCGGTCATGGGACTGGGAGTACAGGTAGCGGCAGCTCTAGTGGCACCTCC

and

TAGCGGCAGCTCTAGTGGCACCTCCATGGCAGAAGGGTCCGTAGCAAGGCAACCTGACTTGTTGACCTGTGATGATGAACCGATTCACATTCCTGGAGCAATTCAACCGCATGGGCTGCTCCTTGCTTTGGCAGCGGACATGACGATCGTCGCCGGCTCCGATAACCTGCCCGAGTTGACGGGCTTGGCGATAGGAGCCCTGATAGGCCGCTCAGCCGCTGACGTATTCGATAGCGAAACGCATAACCGGCTTACAATCGCCTTGGCTGAACCGGGCGCGGCCGTGGGAGCACCGATTACTGTAGGCTTTACAATGAGAAAAGACGCCGGCTTTATCGGGTCATGGCACCGACATGACCAGCTGATTTTCCTGGAATTGGAGCCCCCGCAGCGGGATGTAGCCGAACCACAGGCCTTCTTCCGGCGCACTAACTCCGCAATTAGGAGACTGCAGGCAGCTGAGACTTTGGAATCAGCATGCGCGGCAGCTGCACAAGAAGTCCGGAAAATCACGGGTTTTGACCGAGTCATGATCTATAGATTCGCGAGCGATTTCTCAGGAGAAGTTATTGCGGAAGACCGATGCGCGGAGGTAGAATCTAAGCTTGGGTTGCACTACCCCGCCTCCACCGTTCCGGCGCAAGCCAGACGGCTCTATACCATTAATCCGGTGCGGATCATTCCAGATATAAATTACCGGCCTGTACCTGTGACACCGGATTTGAACCCTGTCACGGGCCGACCGATAGACCTCAGCTTCGCTATATTGCGATCTGTGTCACCGGTCCACCTCGAGTTTATGAGGAATATAGGCATGCATGGTACAATGTCCATTTCCATTCTCCGGGGTGAACGGCTTTGGGGCCTCATCGTTTGTCACCATCGAACACCGTATTACGTCGATCTCGACGGCAGACAGGCATGTGAGTTGGTCGCTCAGGTACTCGCTTGGCAGATAGGGGTAATGGAGGAG

PCR fragments were amplified using the following primers:

PiggyBacPuro backbone fwd: ACCTGCGGGGACGTGGAAGAAAACCCGGGTCCAatgaccgagtacaagcccacggtg,
rev: cattccacagggtcgacagtacaagcaaaaag.
8× TOPFlash fwd: cttgtactgtcgaccctgtggaatgaagtgcaggtgccagaacatttctc,
rev: GTCGGGCCACGCTGCCTTCAGCCATggtggctttaccaacagtaccgg.
tdIRFP1 fwd: ATGGCTGAAGGCAGCGTGGC,
rev: GGAGGTGCCACTAGAGCTGC. tdIRFP2
fwd: TAGCGGCAGCTCTAGTGGCAC,
rev: GGTTTTCTTCCACGTCCCCGCAGGTCAACAAACTTCCGCGACCTTCTCCGCTCCCCTCCTCCATTACCCCTATCTGCCAAGCG

All above constructs were transformed into Top10 competent cells prepared using Mix & Go *E. coli* Transformation Kit and Buffer Set (Zymo Research #T3002), cultured on lysogeny broth agar plates to select for antibiotic resistance using standard workflows for molecular cloning and DNA production (49). Plasmid DNA was purified using the Zyppy Plasmid Miniprep kit (Zymo Research #D0436). In addition to antibiotic selection, constructs were verified via Sanger sequencing using primers targeting fusion junctions of relevant construct domains.

### Lentiviral Production and Transduction.

Production of lentivirus carrying opto-GSK3 was accomplished via cotransfection of pLV_Cry2-tdeGFP-GSK3b, pCMV dR8.91 (obtained from Jared Toettcher’s Lab at Princeton University, Princeton, NJ) and pMD 2.G at a 1:0.88:0.11 mass ratio using standard PEI-based transfection procedures (50). Cells were incubated for 24 h before replacing with fresh media and allowing for lentiviral production for an additional 48 h. Supernatant was harvested, filtered through a 0.22-μm filter and added to plated cells for transduction. Note that all steps for lentiviral production, transduction, and subsequent maintenance of cell lines were carried out in the presence of far-red light or the complete absence of light in an attempt to eliminate the possibility of Cry-2 opto-GSK3 clustering interference with cell growth or virus production.

### Construction of CRISPR gRNA Constructs and Homology-Directed Repair Templates.

Genomic edits in 293Ts were carried out in cells constitutively expressing Cas9 to maximize editing efficiency.

#### pCAB_minimal guide RNA backbone.

A vector expressing guide RNA (gRNA) and Cas9 obtained from M.Z.W. was subcloned to remove the unnecessary Cas9 ORF via PCR using the following primers:

fwd: acgcgccctgtagcg
rev: cttaatgcgccgctacagggcgcgtggtacctctagagccatttgtctgc,

assembled, cloned, purified, and verified as described in the previous section. The baseline pCab_minimal construct was then subsequently used for production of gRNAs targeting exon1 of the human genomic loci of CTNNB1, CSNK1a1, and GSK3B. Primers creating one to three (depending on protospacer adjacent motif site availability/predicted on/off-target editing scores) unique protospacers targeting the 50-bp window surrounding the first codon of each gene were annealed and cloned into the pCAB_minimal via BbsI digestion and ligation (New England BioLabs #R3539S, Takara #6023) using standard protocols (51). The following primers were used for sticky-end ligation of protospacers:

CTNNB1_1 fwd: caccgTGAGTAGCCATTGTCCACGC rev: aaacGCGTGGACAATGGCTACTCA
CTNNB1_2 fwd: caccgTGAAAATCCAGCGTGGACAA rev: aaacTTGTCCACGCTGGATTTTCAc
CTNNB1_3 fwd: caccGCGTGGACAATGGCTACTCA rev: aaacTGAGTAGCCATTGTCCACGC
CSNK1a1_1 fwd: caccGGCCAAGCCCCGACACCTCT rev: aaacAGAGGTGTCGGGGCTTGGCC
CSNK1a1_2 fwd: caccgAGGCTGAATTCATTGTCGGA rev: aaacTCCGACAATGAATTCAGCCT
GSK3B fwd: caccCGAAGAGAGTGATCATGTCA rev: aaacTGACATGATCACTCTCTTCG

AXIN1 gRNAs were ordered complete from IDT. Four different protospacer sequences were used (in separate reactions) with the same homology-directed repair (HDR) template to maximize chance of target locus cutting. Cells from each reaction were then pooled 7 d after transfection and subsequently enriched together.

Protospacer sequences:

AXIN1_1: GGCCGTCCTGCCCGTCTTTG
AXIN1_2: GTCTTTGAGGAGAAGATCAT
AXIN1_3: gTCTTTGAGGAGAAGATCATC
AXIN1_4: GGAGAAGATCATCGGCAAAG.

#### HDR templates.

Blunt-end PCR products were used in conjunction with gRNAs to template genomic edits containing desired knock-ins. Blunt-end, double-stranded HDR templates were created from templates obtained via DNeasy Blood and Tissue genomic prep (Qiagen, 69504) of the 293T cell line to be edited (see next section). PCR was conducted using primers targeting amplicons of a 500- to 1,000-bp window centered on the intended cut site. The following primers were used to amplify genomic loci homology regions:

tdmRuby3: GGcGGTAGTGGcGGcGGAagcATGGTTAGCAAAGGGGAGGAGCTTATAAAGGAAAATATGAGAATGAAAGTTGTCATGGAAGGTTCAGTGAATGGCCATCAGTTTAAATGTACAGGTGAAGGCGAGGGACGCCCTTATGAAGGAGTCCAAACTATGAGGATCAAAGTCATAGAGGGAGGTCCTCTCCCCTTCGCCTTCGATATCCTCGCCACCTCTTTCATGTATGGTTCAAGAACATTTATCAAGTATCCTGCCGATATACCAGACTTCTTTAAGCAGTCATTTCCAGAAGGTTTCACTTGGGAACGAGTCACTAGGTATGAGGACGGCGGGGTTGTGACAGTAACTCAAGACACCTCTTTGGAAGATGGTGAGTTGGTCTACAACGTGAAGGTACGCGGGGTTAATTTCCCTTCTAACGGGCCTGTTATGCAAAAGAAGACAAAGGGTTGGGAGCCAAATACCGAGATGATGTATCCTGCAGATGGTGGCCTGCGGGGCTATACCGACATCGCTCTGAAGGTAGACGGCGGGGGCCACCTCCATTGTAATTTTGTAACCACTTACAGGTCTAAGAAGACCGTGGGTAACATTAAGATGCCAGGGGTTCATGCTGTCGACCATAGATTGGAGCGGATAGAAGAAAGCGACAACGAGACCTACGTCGTGCAACGCGAAGTCGCAGTAGCCAAGTATTCCAATCTCGGGGGAGGTATGGATGAACTCTATAAAGGCGGATCCGGTGGTGTGTCCAAGGGAGAAGAACTGATCAAAGAGAACATGAGGATGAAGGTCGTGATGGAGGGCAGCGTCAACGGACACCAATTCAAGTGCACCGGAGAGGGAGAAGGCAGACCATACGAGGGCGTGCAGACAATGAGAATTAAGGTGATCGAAGGCGGACCACTGCCTTTTGCTTTCGACATTCTGGCTACAAGCTTCATGTACGGCAGCAGGACCTTCATTAAATACCCCGCTGACATCCCTGATTTTTTCAAACAAAGCTTCCCTGAGGGCTTTACCTGGGAGAGAGTGACAAGATACGAAGACGGAGGCGTCGTCACCGTCACACAGGATACAAGCCTGGAGGACGGAGAACTGGTGTATAACGTCAAAGTCAGAGGAGTGAACTTTCCCAGCAATGGCCCCGTGATGCAGAAAAAGACCAAAGGCTGGGAACCTAACACAGAAATGATGTACCCAGCCGACGGAGGACTGAGAGGATACACAGACATTGCCCTCAAAGTGGATGGAGGAGGACATCTGCACTGCAACTTCGTCACAACCTACAGATCCAAGAAAACAGTCGGAAATATCAAGATGCCTGGCGTGCACGCCGTGGATCACAGGCTGGAAAGGATTGAGGAGTCCGATAATGAAACATATGTGGTCCAGAGGGAGGTGGCCGTCGCTAAATACAGCAACCTGGGCGGCGGCATGGACGAGCTGTACAAGGGGGGATCAGGAGGaGGctct
CTNNB1 fwd: ATAAAAAGACATTTTTGGTAAGGAGGAGTTTTCACTGAAGTTCAGCAGTGATGGAGCTGTGGTTGAGGTGTCTGGAGGAGACCATGAGGTCTGCGTTTCA CTAACCTGGTAAAAGAGGATATGGGTTTTTTTTGTGGGTGTAATAGTGACATTTAACAGGTATCCCAGTGACTTAGGAGTATTAATCAAGCTAAATTTAAATCCTAATGACTTTTGATTAACTTTTTTTAGGGTATTTGAAGTATACCATACAACTGTTTTGAAAATCCAGCGTGGACAGGcGGTAGTGGcGGcGGAagc
rev: TAGGGAACCACCTAACAGTTACTCACTGAATCAGTGGAAGAATGGTACTGCATCCAGGCTCCAGAAGCAGTCATCCAGACTAGATTCCTGCTGGTGGCTT GTTTGCTATTTCACCAAGCCATTAGGAGGAGTGAGCAGAAAATGGAGCAAAAGGTAGCCTGACAAGTAAGCAGGGAGAGAGGAAAGCAGGGGGATCTCAGCCAGACTGGCTTAATGGCAACGAAGCAGAGCCCCAATTCAGTAACTAAAGATTTAATGACACAAACCTTGAGTAGCCATagagCCtCCTCCTGATCCCCC

Note that CTNNB1 homology arms were synthesized (requiring no genomic amplification step) and provided as a generous gift from Integrated DNA Technologies.

CSNK1a1fwd: CCAGCCCGCGACGTC rev: CTTGACCCTTTTAGGGAGACAGCG
GSK3B fwd: GATTTGCCCTCTCTTTTCTCTCCTCC rev: CCAAATAAATATCATATTATCTCAATTCAAGGTTAATGAGACCG

The above amplicons were then used in a second round of PCR to obtain separate upstream and downstream homology arms that flanked desired knock-ins, and overlap extension was used to construct the final desired amplicons bearing tdmRuby3 and 7AA GS linker. The following primers were used:

Generic tdmRuby3 insert fwd: GGcGGTAGTGGcGGcGGAagcATGGTTAGCAAAGGGGAGGAGC, rev: agagCCtCCTCCTGATCCCCCCTTGTACAGCTCGTCCATGCC
CSNK1a1 upstream homology arm rev: gctTCCgCCgCCACTACCgCCCCTGAGAGACGAAGATGGAGGC
CSNK1a1 downstream homology arm fwd: GGGGGATCAGGAGGaGGctctATGGCGAGTAGCAGCGGC
GSK3B upstream homology arm rev: gctTCCgCCgCCACTACCgCCGATCACTCTCTTCGCGAATCACC
GSK3B downstream homology arm fwd: GGGGGATCAGGAGGaGGctctATGTCAGGGCGGCCC
AXIN1 upstream homology arm fwd: CTTCACCCACATGTGGTCATTGCAC
AXIN1 upstream homology arm rev: CGGCAAAGTGGAGAAGGTGGACGGcGGTAGTGGcGGcGGAagc
AXIN1 downstream homology arm fwd: GGGGGATCAGGAGGaGGctctTGATAGGCTGGTGGGCTGGCC
AXIN1 downstream homology arm rev: CACCTGAAGCTGGCAGCAGG

Note that original upstream fwd and downstream rev primers listed above for isolating genomic loci were reused in the present step and thus not repeated here.

### CRISPR-Cas9 Fluorescent Tagging.

Bare 293T cells were first cotransfected using polyethyleniminePEI (50) with the H2B-mTagBFP2 vector and Super PiggyBac Transposase-expressing vector (System Biosciences Inc. #PB210PA-1) via polyethylenimine (Sigma #408727-100ML) transfection reagent and standard workflows (50). Cells were allowed 72 h following transfection to reach steady-state expression of integrated construct and were enriched via two rounds of fluorescence-activated cell sorting (FACS, SH800S, Sony Biotechnology) for cells fluorescent in the 450-nm excitation (blue) channel: a bulk enrichment to obtain a largely “positive” population and a second to obtain clonal populations. A high-expressing clone was expanded and used as a “chassis” cell line for subsequent CRISPR editing.

CRISPR chassis cells were then cotransfected with one of the constructed gRNA plasmids and respective HDR templates at a 2:1 HDR template:gRNA plasmid molar ratio and allowed 72 h to reach steady-state expression. Similar to the process described above, cells were subject to two rounds of FACS (561-nm excitation, red laser) to obtain a clonal population. Knock-in validation was accomplished via a combination of fluorescence microscopy, genomic PCR, and sequencing (using primers for initial amplification of loci and construction of HDR templates). In the case of all intended knock-ins, spatiotemporal fluorescence expression of cell populations was binary (either fluorescent or not) and uniform (no detected variation in brightness or localization between fluorescent clones), suggesting that selected clones were broadly representative of overall edited populations.

### Development of Inducible Axin1, APC, and β-Cat Cell Lines.

The 293Ts were cotransfected as described in the previous section with PiggyBac and compatible XLone-Axin-tdmRuby3 and pPig_CuO-APC-tdmIRFP670::CymR expression cassettes. Seventy-two hours after transfection, cells were selected in 1 μM Blasticidin (Invivogen, #ant-bl-05) and 100 μg/mL Hygromycin B Gold (Invivogen, #ant-hg-1). Blast+/Hygro+ cells were then clonally sorted via FACS as described in the previous section to obtain a uniform population for experiments. For iPSCs, Both Piggyback and Donor plasmids were chemically transfected when cells reached 30% confluency using Lipofectamine Stem Transfection Reagent (manufacturer’s protocol). Following transfection, Blasticidin selection (1 μM) was initiated 5 d later. At the end of Blasticidin selection, 12 clones were manually picked under a dissection microscope and continuously cultured in Blasticidin (1 μM) for an additional week. Upon fluorescence signal confirming successful integration, Blasticidin (1 μM) treatment ceased, and one clone was chosen for the remaining experiments.

### Small Molecules.

CHIR 99021 (STEMCELL Technologies #72052) was resuspended in DMSO according to supplied manufacturer recommendations and diluted to 5× concentrated stocks in culture medium immediately prior to use on cells. In all cases, CHIR was used at 10 μM. Doxycycline hyclate (Sigma Aldrich #D9891-1G) was resuspended in phosphate-buffered saline (PBS) and diluted to 5× desired concentration in culture medium prior to use. Stock cumate solution (System Biosciences #QM100A-1) was diluted to 5× in culture medium prior to use.

The “Low” dose of Dox referred to in Fig. 2 *C* and *D* in the context of Axin and APC induction was 20 ng/mL concentration in culture medium, “High” dose was 200 ng/mL. “Low” dose of Cumate was 100 ng/mL, and “High” was 1mg/mL. The dose of Dox used in β-cat induction in *SI Appendix*, Fig. S4 *I* and *J* was 100 ng/mL.

### Wnt-3a Treatments.

Recombinant Human Wnt-3a (R&D Systems 5036-WN-010) was resuspended in in PBS containing 0.1% bovine serum albumin according to supplied manufacturer recommendations and diluted to 5× concentration in culture medium immediately prior to use. In all cases, Wnt-3a was used at a final concentration of 1 μg/mL.

### Antibodies, Immunofluorescence, and Western Blot.

Primary antibodies used for immunofluorescent markers of the centrosome were α-GM130 (BD 610822, 1:1,000 dilution [dil.]) and α-γ-tubulin (Sigma Aldrich T5326-25UL, 1:1,000). The secondary used for both stains was α-Ms-Alexa-488 (Invitrogen A28175, 1:1,000). Tissue fixation and staining was carried out using standard protocols using cold methanol (52). Immunofluorescent samples were imaged using confocal microscopy (see below). Antibodies used for Western blotting and immunofluorescence were α-β-catenin (Cell Signaling, #2698S, 1:1,000) and α-β-actin (Sigma, A3853, 1:1,000). Secondary antibodies used were α-Gt-680RD and α-Ms-800CW (Licor 926-6807 and 926-32212, respectively, both 1:10,000 dil.). Standard immunoblot procedures were used (53).

### Imaging.

All live and fixed cell imaging experiments were carried out using a Nikon W2 SoRa spinning-disk confocal microscope equipped with incubation chamber maintaining cells at 37 °C and 5% CO_2_. Glass-bottom culture plates (Cellvis #P96-1.5H-N) were pretreated with bovine fibronectin (Sigma #F1141) in the case of 293Ts or Matrigel in the case of H9 and iPSCs, and cells were allowed to adhere to the plate before subsequent treatment or imaging. FRAP was performed via custom Nikon NIS Elements JOBs function and 488-nm FRAP laser (Nikon LUN-F laser unit, 100-mW power output from the APC fiber tip).

### Optogenetic Stimulation.

Spatial patterning of light during timelapse fluorescent imaging sessions was accomplished via purpose-built microscope-mounted LED-coupled digital micromirror devices (DMDs) triggered via Nikon NIS Elements software. Stimulation parameters (brightness levels, duration, and pulse frequency) were optimized to minimize phototoxicity while maintaining continuous activation of Cry-2. For DMD-based stimulation on the microscope, the final settings for “Light ON” were 25% LED power (λ = 455 nm), 2-s duration pulses every 30 s. For experiments that did not require frequent confocal imaging, cells were stimulated via a benchtop LED array purpose built for light delivery to cells in standard tissue culture plates (“OptoPlate”) adapted from previously established designs (54). The same light delivery parameters were used for OptoPlate-based stimulation as for microscope-mounted DMDs. Light was patterned to cover the entire surface of intended wells of plates used, rather than a single microscope imaging field.

### Image Analysis.

All quantification of raw microscopy images was carried out using the same general workflow: background subtraction > classification > measurement > normalization > statistical comparison. Subcellular segmentation of nuclear fluorescence was performed via custom Matlab scripts using H2B-mTagBFP2 brightness, size, and circularity to mask objects. When experimental conditions did not permit segmentation via H2B-mTagBFP2 nuclear fluorescence (such as with live-cell optogenetic stimulation), cells were selected at random using custom ImageJ macro that generates random regions of interest (ROIs) (available upon request). Unless otherwise noted, mean fluorescent intensity of ROIs were measured and subsequently processed. Raw measurements were compiled, processed, and plotted via custom Matlab scripts, available upon request.

### Statistical Analysis.

Statistical parameters are provided in Table 1.

**Table 1.** Statistical Parameters

*P* value range	Symbol
0.01 < *P* < 0.05	*
0.001 < *P* < 0.01	**
*P* < 0.001	***

All statistical tests were carried out on final grouped data points presented in figures using independent samples *t* tests (Matlab function “ttest2”) except for *SI Appendix*, Fig. S4*D* which was the result of one-way ANOVAs.

### Simulation Methods.

We used the Python-based FEniCS computing environment (https://fenicsproject.org/) to solve the modified Cahn–Hilliard partial differential equations using the finite element method. In our simulation, we represent the volume fraction of each DC protein, $\phi_{i}$, as an incompressible volume such that $\sum_{i=1}^{N}\phi_{i}=1$ and approximate the reaction rates with spatially dependent analogs to well-mixed reactions using the simplified, non-state-dependent description of the second-order rate $R_{i}=k_{i,j}\phi_{i}\phi_{j}$, with production and consumption denoted by the sign of $k_{i,j}$ (55, 56). The Cahn–Hilliard equation, in its general form, is a parabolic equation with first-order time derivatives, and second- and fourth-order spatial derivatives. To solve this equation using a standard Lagrange finite element basis, the equation is recast as two coupled second-order equations,

$$\frac{\partial \phi_{i}}{\partial t}=\nabla \cdot M\left(\nabla \left(\mu_{i}\right)\right)+R_{i}\left(k_{i,j},\phi_{i},\phi_{j}\right)$$

$$\mu_{i}=\frac{\partial F}{\partial \phi_{i}}-\lambda \nabla^{2}\phi_{i},$$

where $M_{i}$ is the mobility constant, with all DC components having the same diffusion rate, λ is the surface energy parameter that dictates the length of transition regions between domains, and F is the polynomial double-well description of the free energy,

$$F=\overset{N-1}{\underset{i=1}{\sum }}\overset{N}{\underset{j=2}{\sum }}\chi_{i,j}\phi_{i}^{2}\phi_{j}^{2},$$

where $\chi_{i,j}$ describes interaction strength between DC proteins, the cytoplasm, and the centrosome. We modeled centrosomal nucleation as a region in the simulation with increased interaction strength as has been done previously to describe nucleation sites (57). To determine the size of this nucleation region, we measured the relative volume of centrosomally localized DC kinases and β-cat (*SI Appendix*, Fig. S3*B*), and R_i_ is the added reaction term such that

$$R_{i}\left(k_{i,j},\phi_{i},\phi_{j}\right)=k_{i,j}\phi_{i}\phi_{j}\;\text{for}\;\text{the}\;\text{creation}\;\text{of}\;\phi_{i}$$

and

$$R_{i}\left(k_{i,j},\phi_{i},\phi_{j}\right)\;=\;-k_{i,j}\phi_{i}\phi_{j}\;\text{for}\;\text{the}\;\text{consumption}\;\text{of}\;\phi_{i}.$$

The system is time discretized according to established methods (58). Assuming that the total free energy of the system decreases to a minimum with time, we use the built-in Newtonian solver in the FEniCS environment to approximate the forward evolution of the system in time. To represent the enzyme activities in the DC, we model only clients, with scaffolds existing implicitly as the interaction parameters between system components. Representations are given in Table 2.

**Table 2.** Representations of interaction parameters between indicated model system components

Component variable	Component name	CH equation
$\phi_{1}$	GSK3β	$\frac{\partial \phi_{1}}{\partial t}=M_{1}\nabla^{2}\mu_{1}$
$\phi_{2}$	CK1α	$\frac{\partial \phi_{2}}{\partial t}=M_{2}\nabla^{2}\mu_{2}$
$\phi_{3}$	β-cat	$\frac{\partial \phi_{3}}{\partial t}=M_{3}\nabla^{2}\mu_{3}-\left(k_{2,3}\cdot \phi_{2}\cdot \phi_{3}\right)$
$\phi_{4}$	Phospho-S45 β-cat	$\frac{\partial \phi_{4}}{\partial t}=M_{4}\nabla^{2}\mu_{4}+(k_{2,3}\cdot \phi_{2}\cdot \phi_{3})\;-\;(k_{1,4}\cdot \phi_{1}\cdot \phi_{4})$
$\phi_{5}$	Phospho-S33/S37/S45/T41 β-cat	$\frac{\partial \phi_{5}}{\partial t}=M_{5}\nabla^{2}\mu_{5}+(k_{1,4}\cdot \phi_{1}\cdot \phi_{4})$
$\phi_{6}$	Cytoplasm	$\frac{\partial \phi_{6}}{\partial t}=M_{6}\nabla^{2}\mu_{6}$
$\phi_{7}$	Nucleator	$\frac{\partial \phi_{7}}{\partial t}=M_{7}\nabla^{2}\mu_{7}$

### Interaction Parameter.

One of the key factors that tunes system behavior is the interaction parameter χ. Assuming a system with constant temperature and pressure, the interaction parameter determines the free energy of the system. When χ is positive between two components, the system will tend to demix. If χ is negative between two components, they will tend to mix. Lastly, if χ is neutral, the two components are interactionless. For simplicity, we limited interactions to one of three types: binding (χ ≈ −0.1), neutral (χ ≈ 0), and separating (χ ≈ 2). As noted above, we represent the binding action of DC scaffolds implicitly. Scaffold interactions are taken to be of similar strength and were obtained from literature values described in Table 3.

**Table 3.** Binding actions of modeled DC components, provided with citations from which they were obtained

Interaction	Behavior	Source
Scaffold to GSK3β	Binding	Refs. 36 and 42
Scaffold to CK1α	Binding	Refs. 36 and 42
Scaffold to β-cat	Binding	Ref. 42
Scaffold to P1 β-cat	Binding	Ref. 11
Scaffold to P4 β-cat	Neutral	Refs. 11 and 42
Scaffold to cytoplasm	Separating	This study
Scaffold to centrosome	Binding	This study and ref. 31

Given that the APC/Axin interacts with the DC proteins, the following interaction constants were selected for the system with implicit Axin. We set mixing = 2.0, neutral = 0.0, and demixing = −0.1.

### Simulation Flow.

First, all parameters are defined (χ, *λ*, *∂t*, and *M*). We generate a grid mesh with closed boundary conditions to mimic the closed system within a cell. A layer is generated for each simulated component, and ±5% noise of the initial value is added to induce inhomogeneities. The FEniCS package partial differential solver is called to generate the chemical potential with respect to each component. The final step is to define the output file path and then use the built-in Newton solver to generate the simulation. The simulations are then rendered using Paraview software. A detailed Python notebook of the simulations is available on https://github.com/MZWLab/Lach2022.

### Nucleation Efficiency Parameter Scans.

We defined the nucleation efficiency of β-cat processing for a given simulation by comparing the ratio of the integrated P4-β-cat to β-cat between identical simulations with and without a nucleator (*SI Appendix*, Fig. S3*F*). This allowed us to test the sensitivity of a single metric to alterations in our model’s parameters. In Fig. 3*E*, we independently altered the simulated phosphorylation rates of CK1 and GSK3, K1 and K2, to examine how nucleation efficiency was changed. Our findings are intuitive, in that the faster K1 and K2 are, the less nucleation leads to an efficiency gain for the system. In Fig. 3*F*, we examined nucleation efficiency as a function of the free energy of binding between each of the individual DC clients and the cytoplasm, finding that, in general, increasing the free energy penalty of client–cytoplasm mixing drove greater accumulation of clients at the nucleator (Movie S6) and also increased the nucleation efficiency.

## Supplementary Material

Supplementary File

Supplementary File

Supplementary File

Supplementary File

Supplementary File

Supplementary File

Supplementary File

Supplementary File

## Data Availability

ImageJ macros for ROI generation and measurement are available upon request. Raw measurements were compiled, processed, and plotted via custom Matlab scripts, available upon request. Full details on creating pLV_Cry2-tdeGFP-GSK3b are available upon request. A detailed Python notebook of the simulations is available on GitHub at https://github.com/MZWLab/Lach2022 (59). All data used to generate figures and graphs are provided in the present document or supplementary materials.

## Change History

September 1, 2022: Movies S3–S7 have been updated.
