# Supplementary material for: Nucleation of the destruction complex on the centrosome accelerates degradation of β-catenin and regulates Wnt signal transmission
Source: Proc Natl Acad Sci U S A. 2022 Aug 29;119(36):e2204688119. doi: 10.1073/pnas.2204688119 (PMC9457612; doi:10.1073/pnas.2204688119)
Supplement: Supplementary File [file pnas.2204688119.sapp.pdf]

PNAS

Supplementary Information for

Nucleation of the destruction complex on the centrosome accelerates degradation of β-catenin and regulates Wnt signal transmission

Ryan S. Lach[1], Chongxu Qiu[1], Erfan Zeyaei Kajbaf[1], Naomi Baxter[1], Dasol Han[3], Alex Wang[1], Hannah Lock[1], Orlando Chirikian[5], Beth Pruitt[1,2,4], Maxwell Z. Wilson[1,3,4,5,*]
[1]Department of Molecular, Cellular, and Developmental Biology, UCSB
[2]Department of Mechanical Engineering, UCSB
[3]Neuroscience Research Institute, UCSB
[4]Center for BioEngineering, UCSB
[5]Biomolecular Science and Engineering, UCSB
*Corresponding author: Maxwell Z. Wilson
Email: mzw@ucsb.edu

This PDF file includes:

Figures S1 to S4
Legends for Videos S1 to S7

Other supplementary materials for this manuscript include the following:

Videos S1 to S7

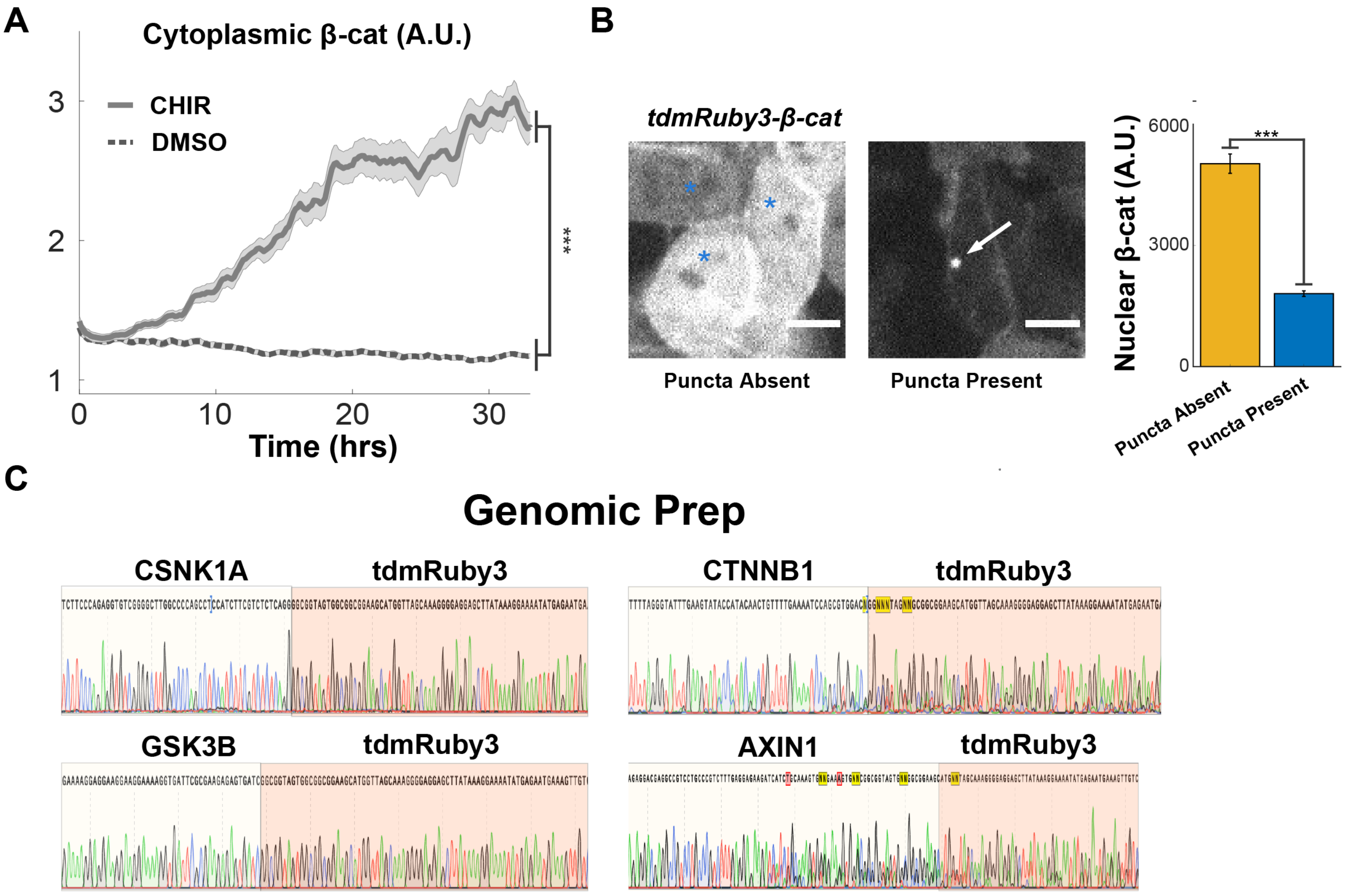

Fig. S1. Endogenously expressed β-catenin puncta are inversely correlated with CHIR-mediated Wnt pathway activation and β-catenin accumulation. A. Measurements of CRISPR cytoplasmic tdmRuby3-β-catenin in live 293Ts, data presented as mean fluorescent intensity fraction of t0 +/- s.e.m. (N = 30 cells per condition). **B.** *Left:* Representative images of tdmRuby3-β-catenin cells +CHIR for 24hrs. Arrows indicate puncta, asterisks indicate puncta absent. *Right:* Comparison of mean nuclear β-catenin fluorescence between +CHIR cells with and without visible β-catenin puncta. **C.** Sanger sequencing traces from genomic PCRs targeting 5' endogenous loci of CRISPR tdmRuby3 knock-ins. Red regions indicate tdmRuby3 insert.

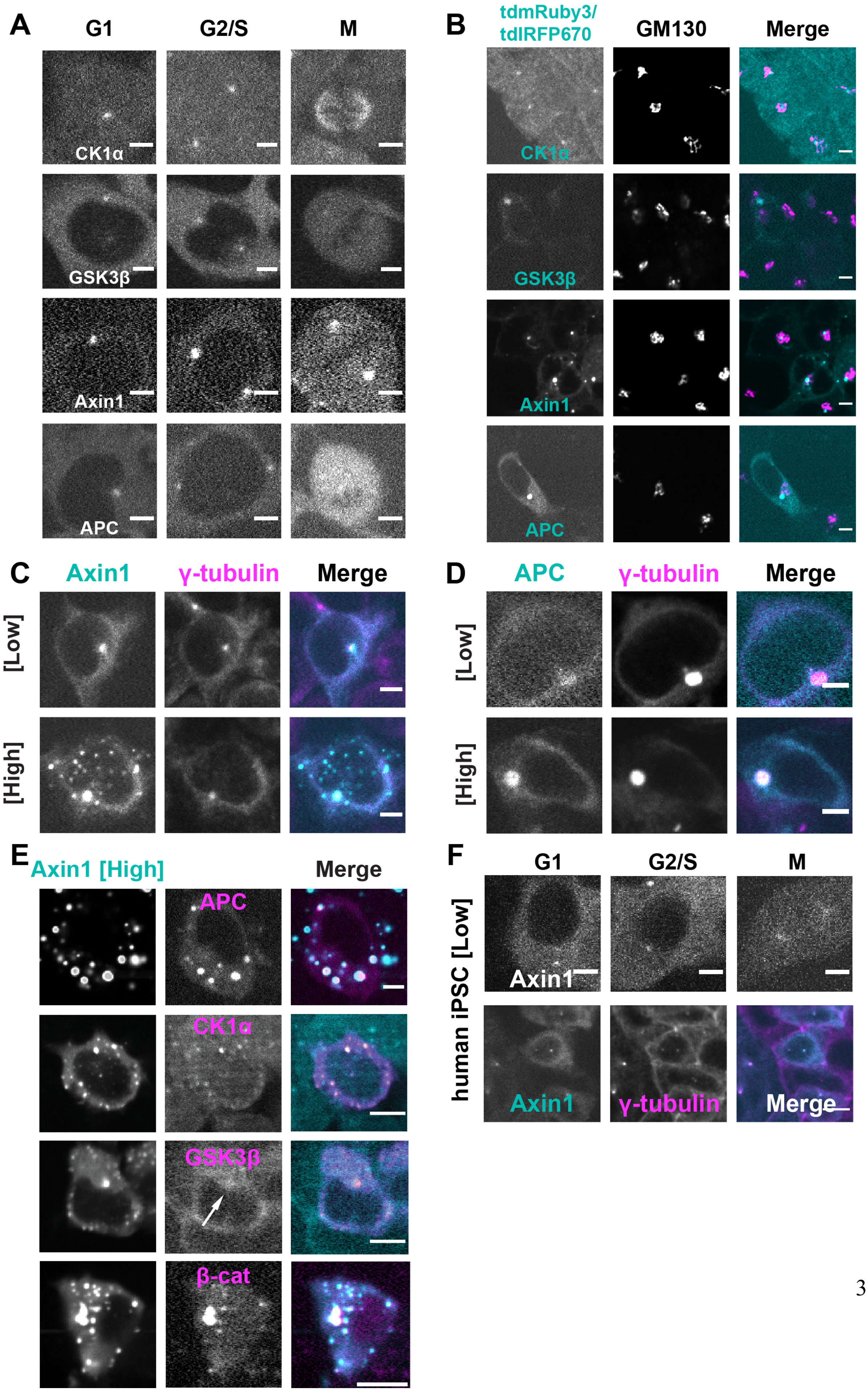
A
G1
G2/S
M
CK1α
GSK3β
Axin1
APC
B
tdmRuby3/
tdIRFP670
GM130
Merge
CK1α
GSK3β
Axin1
APC
C
Axin1
γ-tubulin
Merge
[Low]
[High]
D
APC
γ-tubulin
Merge
[Low]
[High]
E
Axin1 [High]
Merge
APC
CK1α
GSK3β
β-cat
F
G1
G2/S
M
human iPSC [Low]
Axin1
Axin1
γ-tubulin
Merge

Fig. S2. Centrosomal Destruction Complex droplets are spatially correlated with cell cycle progression. A. Representative images of indicated Destruction Complex (DC) components taken live, at various stages of the cell cycle. Montages follow the same cell through time. Scale = 10μm **B.** Representative fixed images of indicated DC components stained for endogenous GM130. Scale = 10μm. **C.** Representative images of fixed cells with varying Axin1 induction and co-stained for endogenous γ-tubulin. **D.** Representative images of fixed cells with varying APC induction and co-stained for endogenous γ-tubulin. **E.** Representative images of live cells bearing indicated proteins co-expressed with high Axin-1; Cumate-induced: APC, CRISPR-integrated: CK1α, GSK3β, Dox-induced: β-cat. Arrow indicates example of depleted centrosomal GSKβ observed under high Axin1 induction **F.** *Upper:* Representative images of live human induced-pluripotent stem cells (iPSCs) at various stages of the cell cycle. *Lower:* Images of fixed human iPSCs co-stained for endogenous γ-tubulin.

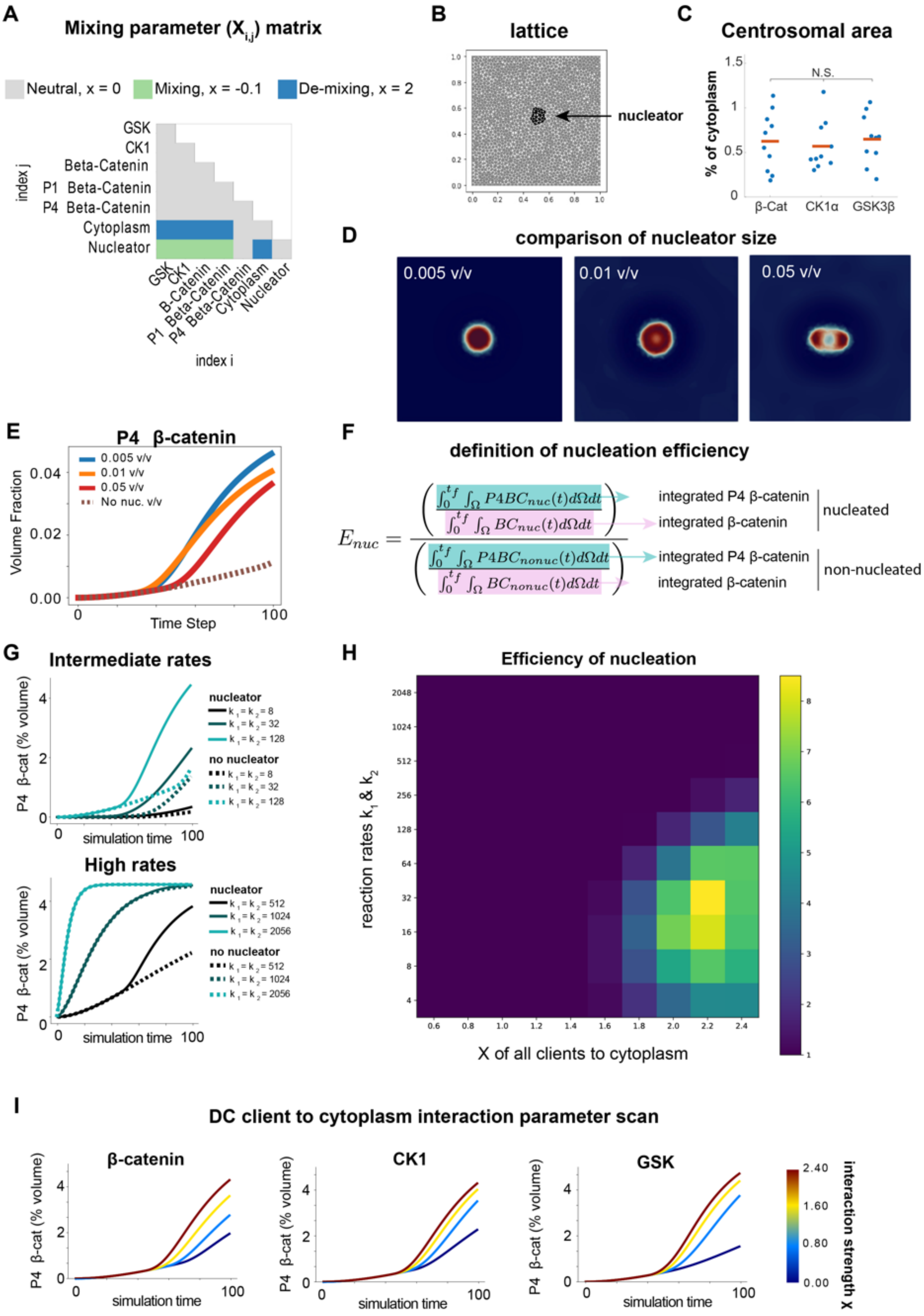

$$E_{nuc} = \frac{\left(\frac{\int_0^{t_f}\int_\Omega P4BC_{nuc}(t)\,d\Omega dt}{\int_0^{t_f}\int_\Omega BC_{nuc}(t)\,d\Omega dt}\right)}{\left(\frac{\int_0^{t_f}\int_\Omega P4BC_{nonuc}(t)\,d\Omega dt}{\int_0^{t_f}\int_\Omega BC_{nonuc}(t)\,d\Omega dt}\right)}$$

Fig. S3. Exploring In Silico Model of Centrosome driven Phase Separation. A. Interaction matrix of each component in the model. Gray indicates a neutral state, blue represents de-mixing, and green represents mixing **B.** Example lattice used to model the system with example nucleator region in black. After initial conditions are assigned, a model of diffusion operates on grid positions based on modified Cahn Hilliard equations. **C.** Quantification of the area of centrosomal droplets in comparison of total cell volume taken from CRISPR-tagged cells. Mean is represented by red line. **D.** Demonstration of the effects of nucleator size on system nucleation process. With a smaller centrosome, the droplet is more densely packed with enzymes whereas a larger centrosome results in droplet separation. **E.** Quantification of the effect of centrosome size on P4 β-catenin generation. **F.** Definition of nucleation efficiency as the ratio of the quotient of P4 β -catenin and β-catenin in a nucleated versus an unnucleated system. **G.** P4 β-catenin accumulation in log2 scan of kinase reaction rates. **H.** Nucleation efficiency of as a function of reaction rates and X (interaction parameter) of all clients and the cytoplasm. **I.** Quantification of in silico models of “opto”-β-catenin, “opto”-CK1, and “opto”-GSK. The graphs show increased gain from “opto”-GSK driven separation.

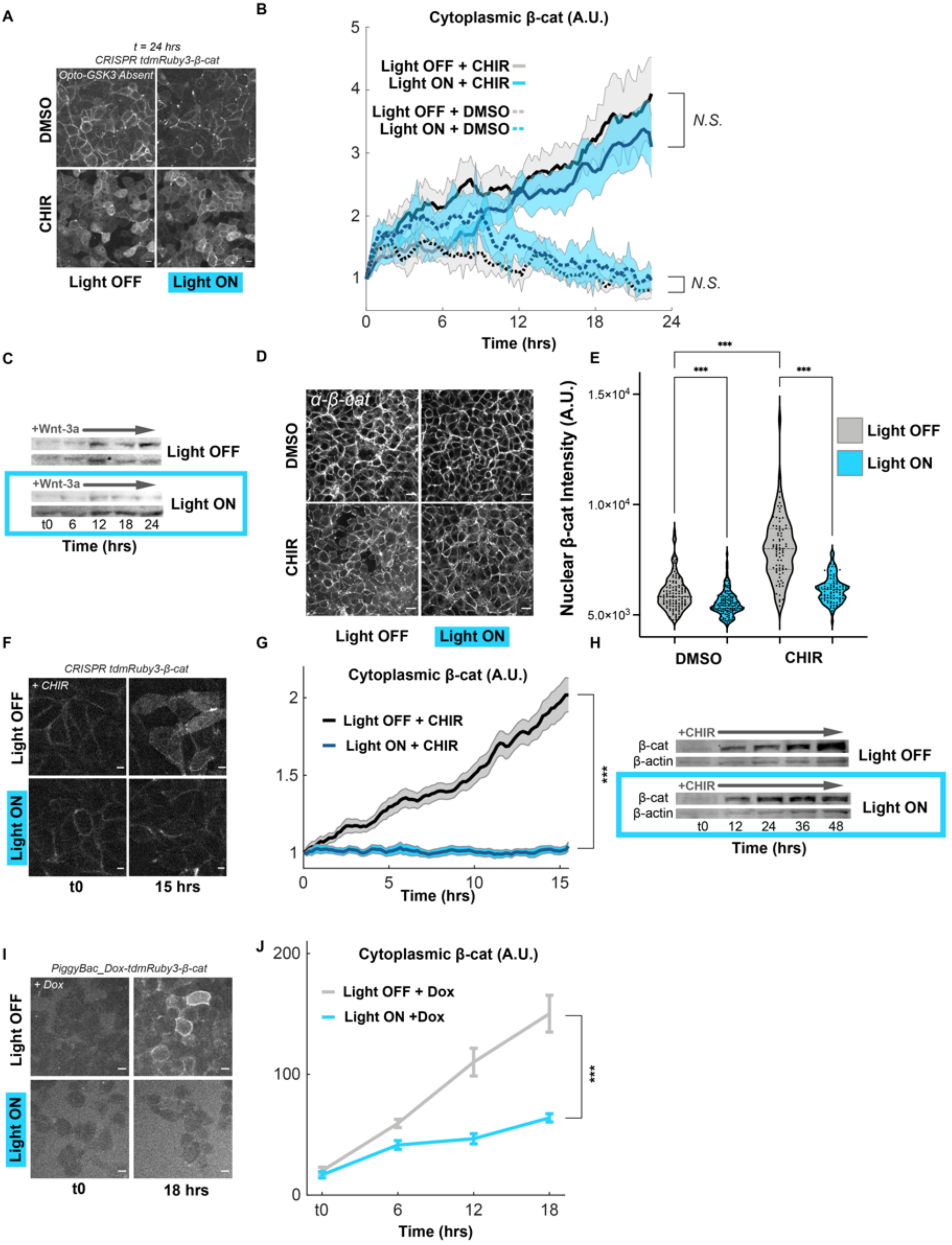
A
t = 24 hrs
CRISPR tdmRuby3-β-cat
Opto-GSK3 Absent
DMSO
CHIR
Light OFF
Light ON
B
Cytoplasmic β-cat (A.U.)
Light OFF + CHIR
Light ON + CHIR
Light OFF + DMSO
Light ON + DMSO
N.S.
N.S.
Time (hrs)
C
+Wnt-3a
Light OFF
+Wnt-3a
Light ON
t0 6 12 18 24
Time (hrs)
D
α-β-cat
DMSO
CHIR
Light OFF
Light ON
E
Nuclear β-cat Intensity (A.U.)
Light OFF
Light ON
DMSO
CHIR
F
CRISPR tdmRuby3-β-cat
+ CHIR
Light OFF
Light ON
t0
15 hrs
G
Cytoplasmic β-cat (A.U.)
Light OFF + CHIR
Light ON + CHIR
Time (hrs)
H
+CHIR
β-cat
β-actin
Light OFF
+CHIR
β-cat
β-actin
Light ON
t0 12 24 36 48
Time (hrs)
I
PiggyBac_Dox-tdmRuby3-β-cat
+ Dox
Light OFF
Light ON
t0
18 hrs
J
Cytoplasmic β-cat (A.U.)
Light OFF + Dox
Light ON +Dox
Time (hrs)

Fig. S4. Opto-GSK3 suppresses β-catenin accumulation due to GSK3β inhibition or exogenous chemical induction. A. Representative images of live cells treated with CHIR or DMSO vehicle, with or without blue light stimulation. Scale = 10μm. **B.** Measurements of experiment shown in A. Data presented as mean +/- s.e.m. (N=30 cells per condition). **C.** Representative Western blots of lysates from 293Ts bearing Opto-GSK3 and treated with Wnt-3a, with or without blue light stimulation for the indicated time course. **D.** Representative images of cells bearing Opto-GSK3 fixed and stained for endogenous β-catenin after culture in the indicated conditions for 48 hrs. **E.** Violin plots of cells from D. **F.** Representative images of 293Ts bearing Opto-GSK3 and endogenously-expressed tdmRuby3-β-catenin treated with CHIR, with or without blue light stimulation for 24hrs. **G**. Measurements from experiment shown in F., lines represent fold-change from t0 means +/- s.e.m. for cells in each condition (Light ON N=67, Light OFF N=50 cells). **H.** Representative Western blots of lysates from 293Ts bearing Opto-GSK3 and treated with CHIR, with or without blue light stimulation for the indicated time course. **I.** Representative images of 293Ts bearing Opto-GSK3 and Dox-inducible -β-catenin-tdmRuby3 treated with Dox, with or without blue light stimulation. Scale = 10μm. **J.** Quantification of experiment in I: lines represent absolute means +/- s.e.m for cells in each condition (Light ON N=28, Light OFF N=27 cells).

Video S1. Cells with β-cat Puncta Resist β-cat Accumulation in response to CHIR. *Left:* Imaging fields of live tdmRuby3-β-cat cells treated with DMSO control. *Right:* Imaging fields of tdmRuby3-β-cat cells treated with CHIR. Arrows indicate β-cat puncta.

Video S2. Activation of Cry-2-Lrp6c Induces β-cat Accumulation *Left*: Imaging fields of live, unstimulated tdmRuby3-β-cat, Cry2-Lrp6c cells. *Right:* Imaging fields of tdmRuby3-β-cat, Cry2-Lrp6c cells stimulated with blue light throughout indicated timecourse. Videos were taken from cells in the same well.

Video S3. Activation of Cry-2-Lrp6c Results in Dissolution of β-cat Puncta Zoomed videos of cells presented in **Supp. Vid. 2.** Arrows indicate β-cat puncta.

Video S4. In-silico behavior of destruction components with a centrosomal region. In-silico model of phase separation behavior for every component involved in a hypothetical WNT pathway over 100 simulation time steps in the presence of a centrosome.

Video S5. In silico behavior of destruction components without a centrosomal region. In-silico model of phase separation behavior for every component involved in a hypothetical WNT pathway over 100 simulation time steps without the presence of a centrosome.

Video S6. Impact of interaction parameter χ on destruction complex component behavior. In-silico model of the destruction components (CK1α, GSK3β, and β-catenin) at various interaction parameter values (χ) over 100 simulation time steps showing that increasing χ increases separation propensity.

Video S7. Activation of Opto-GSK3 Increases Centrosomal Condensate Partitioning. Zoomed video of Opto-GSK3 cells stimulated with blue light throughout indicated timecourse.
